# The *iucC* gene is a major contributor to the virulence of hypervirulent *Klebsiella pneumoniae*

**DOI:** 10.3389/fcimb.2025.1742219

**Published:** 2026-01-12

**Authors:** Chong Chen, Li Wang, Shuo Zu, Hui Ning, Liran Song, Jianbao Dong, Changjiang Sun, Xin Feng, Wenyu Han, Yalu Ji, Jingmin Gu

**Affiliations:** 1 State Key Laboratory for Diagnosis and Treatment of Severe Zoonotic Infectious Diseases, Key Laboratory of Zoonosis Research of the Ministry of Education, Institute of Zoonosis, and College of Veterinary Medicine, Jilin University, Changchun, China; 2 College of Materials Science and Engineering, Jilin Jianzhu University, Changchun, China; 3 College of Veterinary Medicine, Qingdao Agricultural University, Qingdao, China; 4 Jiangsu Co-Innovation Center for the Prevention and Control of Important Animal Infectious Diseases and Zoonoses, Yangzhou University, Yangzhou, China

**Keywords:** aerobactin, hypervirulent *Klebsiella pneumoniae*, *iucC* gene, pig, virulence

## Abstract

**Background:**

Hypervirulent *Klebsiella pneumoniae* (hv*Kp*) is a critical pathogen that causes highly lethal invasive infections with its virulence linked to that of aerobactin, the core siderophore system of hv*Kp*.

**Methods:**

Here, 20 porcine-derived *K. pneumoniae* strains were subjected to capsular genotyping and the virulence of the strains was evaluated through mouse lethality assays. Whole-genome sequencing and analysis was performed on two K2 serotype strains (including highly and weakly virulent strains), and the differential virulence genes were analyzed. The virulence-related gene *iucC*, identified in the hypervirulent K2 serotype strain KP10, was knocked out using homologous recombination to preliminarily explore its function in *K. pneumoniae*.

**Results:**

In this study, a highly virulent K2-type strain KP10 and a low virulent K2-type strain KP6 were screened from 20 strains of porcine *K. pneumoniae*. The results of the genome and virulence gene analyses revealed that compared with KP6, the highly virulent strain KP10 specifically encodes the *iucC* gene. The △ *iucC* mutant of the high-virulence strain KP10 exhibited significantly reduced biofilm formation, decreased siderophore production, moderate serum sensitivity, and attenuated virulence. Furthermore, the mutant displayed decreased adhesion to IPEC-J2 cells, reduced cytotoxicity, and decreased cell mortality. These results revealed that *iucC* is a major contributor to virulence in the KP10 hv*Kp* strain. Notably, *iucC* genes were detected in the genomes of *K. pneumoniae* from humans and six different animal and environmental sources, and some *iucC* genes were shared in *K. pneumoniae* from these sources.

**Conclusion:**

Overall, this work provides a crucial theoretical foundation for elucidating *iucC* function and the pathogenesis of *K. pneumoniae.* Its widespread presence in different hosts and environments considerably increases the risk of cross species transmission and public health events.

## 1 Introduction

*Klebsiella pneumoniae* (*K. pneumoniae*), a gram-negative and rod-shaped bacterium, is part of the Enterobacteriaceae family (Feldman et al., 2019). It is an opportunistic pathogen commonly found in diverse environments including water, soil, plants, and animals and may be part of the normal flora of the human gastrointestinal tract (Choby et al., 2020; Lei et al., 2024). *K. pneumoniae* can cause a variety of nosocomial infections, including pneumonia, urinary tract infections, bloodstream infections, meningitis, and bacteraemia, such as in people with diabetes or malignancies (Wang et al., 2020; Yang et al., 2022). Since the mid-1980s, a new “hypervirulent” *K. pneumoniae* (hv*Kp*) with hypermucoviscosity has been increasingly reported worldwide, particularly in Asia, and has emerged as a clinically significant pathogen (Russo et al., 2018). Unlike “classic” *K. pneumoniae* (c*Kp*), this strain causes highly invasive infections, such as pyogenic liver abscesses, osteomyelitis, and endophthalmitis, predominantly, in younger and healthier people (Lee et al., 2017).

*K. pneumoniae* not only poses a significant threat to human health but also presents considerable risks to animals, particularly those in the swine industry. However, this pathogen typically does not cause fatal infections in pig populations and often manifests as sporadic cases, with only a few severe infections, leading to its pathogenicity being frequently overlooked (Wu et al., 2023). In recent years, with the emergence of hv*Kp* and carbapenem-resistant *K. pneumoniae (*CR*Kp)* on pig farms, infection rates have shown an increasing trend. These strains can not only cause pneumonia in pigs but also lead to sepsis, mastitis, and urinary tract infections (Bidewell et al., 2018; Hou et al., 2024; Yang et al., 2019b). Owing to the widespread transmission of *K. pneumoniae* between animals and humans (Hetland et al., 2025), in-depth research into the pathogenic mechanisms of pig*-*derived *K. pneumoniae* is of significant practical importance for safeguarding public health and enhancing biosecurity management in the swine industry.

The pathogenicity of *K. pneumoniae* is attributed to its multiple virulence factors, including capsules, lipopolysaccharides, flagella, and siderophores. These factors enable bacteria to adhere to host tissues, evade the immune system, and cause tissue damage (Holden et al., 2016). *K. pneumoniae* can secrete four types of siderophores, namely, aerobactin, salmochelin, enterobactin, and yersiniabactin, which have been shown to strongly correlate with *in vivo* virulence and differentiate hv*Kp* strains from c*Kp* strains (Arato et al., 2021; Mendes et al., 2023). These siderophores enable bacteria to acquire iron in iron-limited environments, such as the human host. Among these siderophores, aerobactin, a citrate-hydroxamate siderophore, is critically important for the virulence of pathogenic enteric bacteria. Aerobactin is encoded by four biosynthetic enzymes (“iron uptake chelates” *iucA*, *iucB*, *iucC* and *iucD*) and a transmembrane transporter (“iron uptake transporter” *iutA*) operon, which are involved in the biosynthesis and transport of aerobactin glycosides (Pu et al., 2023). *IucC* is a member of a family of nonribosomal peptide synthetase-independent siderophore (NIS) synthetases that play crucial roles in the biosynthesis of aerobactin (Bailey et al., 2018). Current studies have shown that the *iucC* gene plays a significant role in the pathogenicity of the avian pathogen *Escherichia coli* (APEC) E058 (Ling et al., 2013). Additionally, research has indicated that the synthesis of aerobactin is crucial for the virulence of human derived hv*Kp* (Russo and Gulick, 2019). However, studies on the role of the *iucC* gene in the pathogenicity of hv*Kp* are relatively limited. In particular, the contribution of the *iucC* gene to the virulence of hv*Kp* from animal sources has not been reported.

In this study, we conducted capsular genotyping on 20 porcine-derived *K. pneumoniae* strains and evaluated them through mouse lethality assays. Two K2- serotype strains with varying virulence (including highly and weakly virulent strains) were selected for whole-genome sequencing, followed by the prediction of homologous genes and virulence factor genes. The virulence-associated gene *iucC* was identified in the K2 serotype hv*Kp* KP10. Using homologous recombination technology, we deleted the *iucC* gene and preliminarily explored its function in *K. pneumoniae*.

## 2 Materials and methods

### 2.1 Animals and ethics statement

The animal experiments were approved by the Laboratory Animal Welfare Ethics Committee of Jilin University (project license number: SY202412013). Six- to eight-week-old C57 female mice were purchased from Liaoning Changsheng Biotechnology Co., Ltd. (Shenyang, China. During the experimental period, the temperature of the animal room was controlled with light-dark cycles. The mice were allowed free access to food and water before and after inoculation.

### 2.2 Bacterial strains and culture conditions

Bacterial strains were isolated from pig feces from 4 different pig farms and were confirmed to be *K. pneumoniae* by 16S rDNA sequence amplification and sequencing, along with PCR amplification of the *khe* gene as described in a previous study (He et al., 2016; Wu et al., 2021). All *K. pneumoniae* were routinely cultured using Luria Bertani (LB) (Becton, Dickinson and Company, Franklin Lakes, NJ, USA) at 37°C with shaking at 180 rpm.

### 2.3 Characterization of capsular genotyping and hypermucoviscous phenotype

All the isolated *K. pneumoniae* were typed by wzi gene sequencing according to Aboulela et al (Aboulela et al., 2020). The strains were amplified by PCR using wzi gene specific primers (**Supplementary Table S1**) and the PCR products were subsequently sequenced by Sangon Biotech. The primer sequences can be found in **Supplementary Table S1**.

For the string test to identify the hypermucoviscous phenotype, all strains were inoculated onto agar plates containing 5% sheep blood as described previously to determine whether they exhibited a hypermucoviscous phenotype (Gu et al., 2018). The results of the string test were considered positive when a sticky string longer than 5 mm could be produced by touching and pulling individual colonies upwards with a standard inoculation ring.

### 2.4 Mouse lethality test

The *in vivo* virulence of *K. pneumoniae* was determined, as described by Hu et al (Hu et al., 2023). Twenty strains of *K. pneumoniae* were cultured to the logarithmic growth stage, washed three times with PBS, and diluted to concentrations of 1 × 10^3^ CFU/mL, 1 × 10^4^ CFU/mL, 1 × 10^5^ CFU/mL, 1 × 10^6^ CFU/mL and 1 × 10^7^ CFU/mL. The mice were challenged by intraperitoneal injection (10 mice per group). The infected mice were subsequently monitored daily for 7 days, after which the survival rate of the mice was recorded.

### 2.5 Genome sequencing and annotation

The genome sequencing of *K. pneumoniae* KP10 and KP6 was performed at Beijing Novogene Bioinformatics Technology Co., Ltd., using PacBio RS II. Genomic gene structures were predicted using Prokka. Key genomic characteristics, including genome size, GC content, and the number of coding genes for *K. pneumoniae* KP6 and KP10, were statistically analyzed and visualized using TBtools software. To identify virulence-associated genes, the protein sequences of KP6 and KP10 were aligned against the Virulence Factor Database (VFDB). Using ABRicate (version 1.0.1) with VFDB as the reference database, potential virulence factor-coding genes were screened with default identity and coverage thresholds (≥80% for both identity and coverage). All the annotation results were manually verified to ensure accuracy (Fu et al., 2024).

### 2.6 Construction of the gene deletion and complementation strains

A mutant of the *iucC* gene of *K. pneumoniae* KP10 was constructed with a homologous recombination system according to a previous study (Li et al., 2023). The primers for constructing △ *iucC* and C-*iucC* are listed in **Supplementary Table S1**. To construct the △ *iucC* mutant, upstream and downstream flanking regions were amplified with the primer pairs *iucC*-up-F (*Xba*I)/*iucC*-up-R and *iucC*-down-F/*iucC*-down-R (*Sac*I), respectively. These fragments were subsequently fused by PCR using the primers *iucC*-up-F (*Xba*I) and *iucC*-down-R (*Sac*I). The fusion product was digested and ligated into *Xba*I/*Sac*I-linearized pRE112, creating the suicide plasmid pRE112-*iucC*. This plasmid was introduced into KP10 via conjugation using *E. coli* WM3064 as the donor. The first crossover was selected with ampicillin and chloramphenicol, and the second crossover and plasmid excision were selected with ampicillin and 12% sucrose (Yang et al., 2019a). The △ *iucC* mutant was confirmed by PCR with the primers *iucC*-I-F/*iucC*-I-R and sequencing.

For the complementary strain C-*iucC*, the *iucC* gene including its promoter was amplified with *iucC*-P-F/*iucC*-P-R and cloned and inserted into pBBR1-MCS, generating pBBR-*iucC*. This plasmid was transformed into the △ *iucC* mutant and C-*iucC* was identified by PCR analysis and DNA sequencing. Genetic stability was assessed for △ *iucC* and C-*iucC* using PCR with the primers *iucC*-up-F/*iucC*-down-R and *iucC*-C-F/*iucC*-C-R, respectively.

### 2.7 qRT-PCR of *iucB* and *iucD*

Total RNA was extracted from 5 mL of overnight cultures of KP10 and △ *iucC* strains using TRIzol reagent (TaKaRa, Dalian, China) according to the manufacturer’ s instructions. Reverse transcription was performed using 1 μg RNA in each sample by the Prime Script™ RT Master Mix Kit (TaKaRa, Dalian, China). qRT-PCR analysis was performed with the TB Green PCR Master Mix (TaKaRa, Dalian, China). The primers for qRT-PCR analysis are listed in **Supplementary Table S1**. The 2−ΔΔCt method was used to determine the relative expression level of each gene (Li et al., 2022). 16S rRNA was used as the internal reference gene for internal normalization. The experiments were performed at least three times, and the data were statistically analyzed.

### 2.8 Mucoviscosity assay

The strains KP10, △ *iucC* and C-*iucC* were inoculated onto agar plates with 5% sheep blood for the string test to identify the hypermucoviscous phenotype as described in previous studies (Walker et al., 2019). The viscosity of the supernatant was assessed by low-speed centrifugation of liquid cultures as previously described (Muner et al., 2024). Briefly, the strains were incubated for 6 h and then the cultures were standardized to 1 OD_600_/mL and centrifuged at 1,000 × *g* for 5 min. The OD_600_ value of the supernatant was measured. The experiment was repeated three times.

### 2.9 Bacterial growth experiments

A bacterial growth assay was performed according to a previous study with some modifications (Zhu et al., 2023). Specifically, the strains KP10, △ *iucC* and C-*iucC* were cultured in LB broth overnight. For the iron depleted and replete conditions, the overnight strains were inoculated with 1% LB supplemented with a source of ferrous iron (FeSO_4_) or with the iron chelator 2,2’-dipyridyl to a final concentration of 200 μM and incubated for 24 h at 37°C, after which they were collected at intervals of 1 h and recorded at OD_600nm_. The experiment was repeated three times.

### 2.10 Biofilm formation assay

Biofilm formation was assessed using a microtiter plate biofilm assay according to a previous study with minor modifications (Shao et al., 2020). Specifically, the diluted bacterial cultures (KP10, △ *iucC* and C-*iucC*) were dispensed into a 96-well microplate and subjected to static cultivation at 37°C for 36 hours. The biofilm formation capacity of each strain was subsequently determined by measuring the OD_590_. However, when OD_590C_<OD_590_ ≤ 2OD_590C_, 2OD_590C_<OD_590_ ≤ 4OD_590C_ and OD_590_>4OD_590C_, the biofilm formation ability was judged as weak, moderate and strong, respectively.

### 2.11 Chrome chrome azurol S assay

A chrome azurol S (CAS) assay was used to determine the iron-chelating ability of the bacterial supernatants according to a previous study with some modifications (Holden et al., 2018). Briefly, overnight cultures of KP10, △ *iucC*, and C-*iucC* strains were centrifuged. The supernatant was mixed with an equal volume of CAS assay solution and incubated in the dark for 30 min, after which the absorbance (As) at OD_630_ nm was measured. A mixture of LB medium and CAS solution served as the blank control (Ar). Each sample was assayed in triplicate. Siderophore production was calculated as follows: (Ar – As)/Ar × 100%.

### 2.12 Serum sensitivity assay

The serum sensitivity of strains KP10, △ *iucC* and C-*iucC* was determined as previously described (Al-Busaidi et al., 2024). Briefly, serum was isolated from healthy pig blood by centrifugation. Bacteria (1 × 10^6^ CFU/mL) were incubated with 75% serum at 37°C for 3 hours. Viable colony counts were determined at 0, 1, 2, and 3-hour intervals. On the basis of the results, each strain was classified in triplicate assays as highly sensitive (grade 1–2), intermediately sensitive (grade 3–4), or resistant (grade 5–6).

### 2.13 Mouse infection assay

The *in vivo* virulence of KP10, △ *iucC*, and C-*iucC*, was tested in a mouse model of intraperitoneal infection as described in a previous study with minor modifications (Hu et al., 2022). Female C57 mice were randomized into 4 groups (A–D), with 10 mice per group. Specifically, the mice in group A were treated with 100 μL of KP10 (group A: 1 × 10^5^ CFU), the mice in group B were treated with 100 μL of △ *iucC* (group B: 1 × 10^5^ CFU), the mice in group C were treated with 100 μL of C-*iucC* (group C: 1 × 10^5^ CFU), and the mice in group D were treated with 100 μL of PBS. The mental state and mortality rate of the mice were monitored every 12 h for a period of 7 consecutive days, after which the survival rate was calculated.

Blood and organized bacterial loads were determined. Female C57 mice were randomly allocated into four groups (A–D). Specifically, groups A to C each consisted of 9 mice: group A received a single 100 μL administration of KP10 (1×10^6^ CFU/per mouse), group B was given a single 100 μL dose of △ *iucC* (1×10^6^ CFU/per mouse), and group C received a single 100 μL treatment of C-*iucC* (1×10^6^ CFU/per mouse). Group D, comprising 3 mice, was administered a single 100 μL dose of PBS. The mice were euthanized by an intraperitoneal injection of 100 mg/kg sodium pentobarbital overdose at 3, 6, and 12 h after infection. Blood, liver, spleen, kidneys, and lungs were collected aseptically. The tissues were homogenized in PBS, and both the homogenates and blood were serially plated on LB agar for bacterial enumeration (CFU counting). For histopathology, a separate set of mice was euthanized at 12 h post-infection. Their organs were fixed in 4% formalin, processed, and subjected to hematoxylin and eosin staining for microscopic examination.

### 2.14 Cell adhesion assays

Adhesion assays were performed mainly according to methods described previously (Hsieh et al., 2016). Briefly, IPEC-J2 monolayers (1×10^5^ cells/well in 12-well plates) were infected with *K. pneumoniae* at a multiplicity of infection (MOI) of 100 in serum-free DMEM for 2 h at 37°C with 5% CO_2_. Following incubation, nonadherent bacteria were removed by washing with PBS. Cells were then lysed with 0.2% Triton X-100, and internalized bacteria were quantified by plating lysates on LB agar for CFU counting. The experiments were performed in triplicate.

### 2.15 Cytotoxicity to IPEC-J2 cells

This test was performed to determine the effects of *K. pneumoniae* KP10, △ *iucC* and C-*iucC* on IPEC-J2 cell infection (Wang et al., 2023). Briefly, IPEC-J2 cells in 96-well plates were infected with *K. pneumoniae* (MOI=100) in serum-free DMEM for 2, 4, and 6 h. After they were washed with PBS, the cells were treated with 1% dual antibiotics to kill the extracellular bacteria. Cell viability was then assessed using the CCK-8 assay by measuring the OD_450_ after 2 h of incubation. All the assays were performed in triplicate.

### 2.16 Assessment of cell survival or death by propidium iodide single-staining assay

The viability of the cells was determined using a PI dye and flow cytometer according to previously described methods with minor modifications (Edwards et al., 2007). Briefly, IPEC-J2 cells were infected with *K. pneumoniae* (MOI=100) for 6 h. The cells were then trypsinized, collected by centrifugation, and fixed. After they were washed with PBS, the cells were stained with propidium iodide (PI) in the dark for 30 min and analyzed by flow cytometry (CytoFLEX). The experiments were performed in triplicate.

### 2.17 Phylogenetic tree analysis of the *iucC* gene

Identification of the sequence of the virulence gene *iucC* on the basis of 20 porcine *K. pneumoniae* genomes in this study. In addition, the virulence gene *iucC* sequence was identified from 30,758 publicly available *K. pneumoniae* genomes (integrity > 90%, contamination<5%) retrieved from the NCBI database using BLASTn (v2.16.0+). Multiple sequence alignment of all the identified *iucC* gene sequences was performed using MAFFT (v7.525). Phylogenetic trees were constructed with RaxML (v1.2.2) and visualized using iTOL (v7.1) (Ibrahim et al., 2023).

### 2.18 Statistical analysis

Statistical analysis and data visualization were performed using SPSS 16.0 software for one-way ANOVA and GraphPad Prism 8.0.2, respectively. Data throughout the text and figures are presented as the means ± standard errors of the means. In all analyses, statistical significance was set at *P*<0.05, *P*<0.01, or *P*<0.001.

## 3 Results

### 3.1 Isolation and identification of *K. pneumoniae* strains

In total, 20 isolates of putative *K. pneumoniae* were isolated from pig feces samples. The amplicon sizes of 16S rDNA and *khe* of the isolated strains were approximately 1500 bp and 428 bp, respectively, according to gel electrophoresis (**Supplementary Figure S1A** and **S1B**). BLAST analysis revealed that the 16S rDNA sequences of these strains were 99% identical to those of *K. pneumoniae* strains previously identified in the GenBank database (accession number: CP152779.1).

### 3.2 Determination of the capsular genotyping and hypermucoviscous phenotype

In total, 11 serotypes were obtained from 20 K*. pneumoniae* strains, namely K1, K2, K10, K19, K26, K28, KL46, K54, K80, KL81 and KL116. Among these, the KP10 and KP6 strains belonged to the K2 serotype. Our results indicated that two isolates, KP10 and KP11 had a string longer than 5 mm (**Supplementary Figure S1C**), indicating a hypermucoviscous phenotype.

### 3.3 Toxicity determination of *K. pneumoniae in vivo*

**Figure 1:**
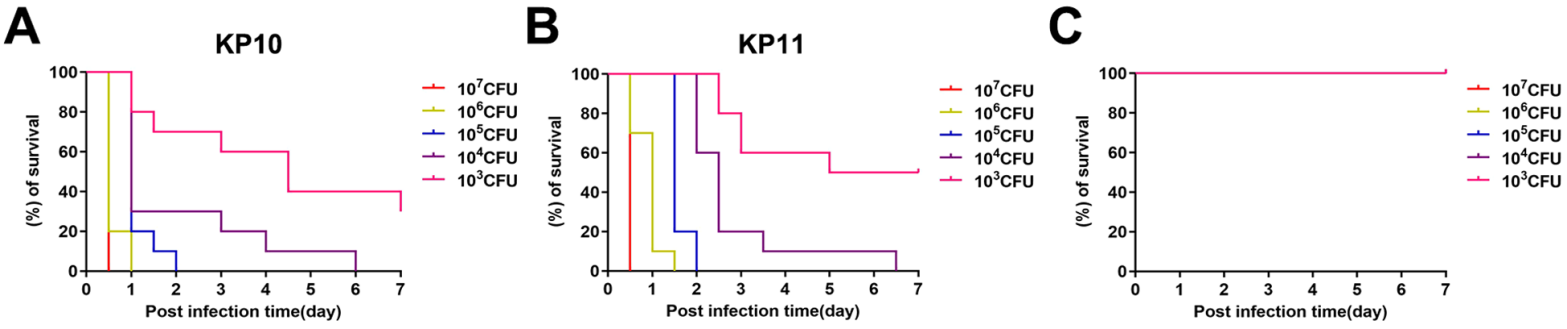
Assessment of the virulence potential of various *K. pneumoniae* strains in a mouse infection model. **(A, B)** Virulence assay of *K. pneumoniae* strains, including KP10 and KP11. **(C)** Virulence assay of the remaining 18 K*. pneumoniae* strains (KP1, KP2, KP3, KP4, KP5, KP6, KP7, KP8, KP9, KP12, KP13, KP14, KP15, KP16, KP17, KP18, KP19 and KP20). The effects of 1 × 10^3^ to 1 × 10^7^ colony-forming units of each *K. pneumoniae* strain on survival were assessed in mice.

As shown in **Figure 1A** and **Figure 1B**, the intraperitoneal injection of 10^3^ CFU/mouse KP10 and KP11 resulted in the complete death of the infected mice within 2 days. In contrast, the remaining 18 strains of *K. pneumoniae* were unable to kill infected mice even at 10^7^ CFU/mouse (**Figure 1C**).

### 3.4 General genomic characteristics

**Figure 2:**
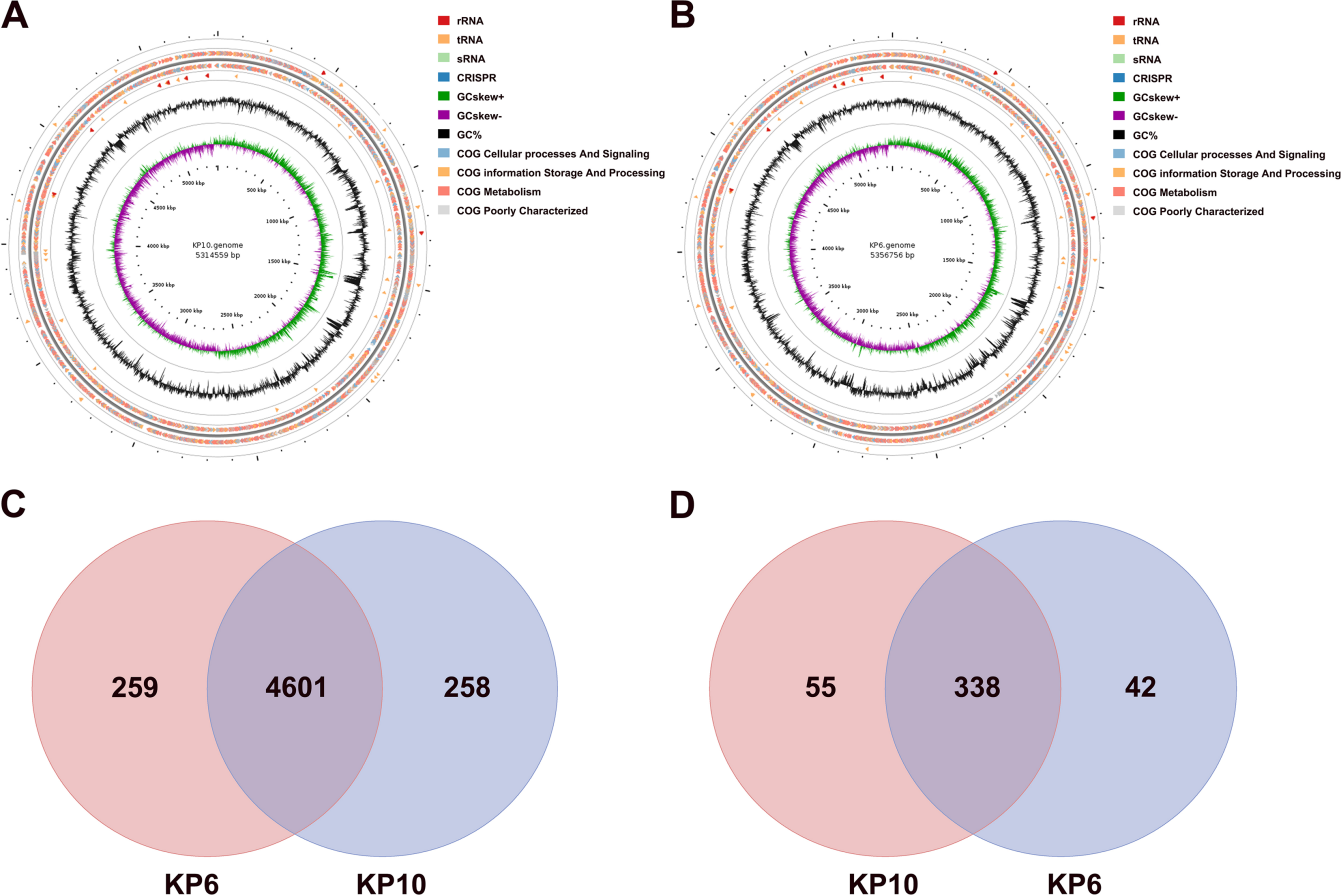
Completed genome maps of the KP10 and KP6 strains. **(A, B)** are the genome maps of KP10 and KP6, respectively. From the inside to the outside, the density profiles of the first ring indicate the GC offset of the positive and negative stranded structures of the genome, the density profiles of the second ring indicate the GC content of the sequences, and the colour blocks in the outermost ring indicate the position of the coding sequences. **(C)** Venn diagram of shared and unique gene families in highly virulent and low virulent strains. **(D)** Venn diagram of shared and unique virulence factor genes in highly virulent and low virulent strains.

Our results revealed that a highly virulent K2-type strain KP10 and a low virulent K2-type strain KP6 were screened from 20 strains of porcine *K. pneumoniae*. Therefore, we performed whole-genome sequencing on these two strains to compare virulence-related genes. The complete genome sequences of the *K. pneumoniae* KP10 and KP6 are available in GenBank. KP10 has a genome size of 5,569,845 bp and KP6 has a genome size of 5,718,111 bp (**Figures 2A, B**). The prediction of homologous genes indicated that *K. pneumoniae* KP10 and KP6 share 4601 coding genes, with KP10 containing 258 unique genes and KP6 containing 259 unique genes (**Figure 2C**). The prediction of virulence factors revealed that KP10 and KP6 contain 393 and 380 virulence factor genes, respectively, with 338 shared virulence factor genes. Additionally, KP10 has 55 unique virulence factor genes, whereas KP6 has 42 unique virulence factor genes (**Figure 2D**). Additionally, we identified relevant genes such as *iroB*, *iucA*, *peg-344*, *rmpA*, and *rmpA2* on the whole-genome plasmid of the KP10 strain.

### 3.5 Construction and identification of the △ *iucC* and C-*iucC* strains

**Figure 3:**
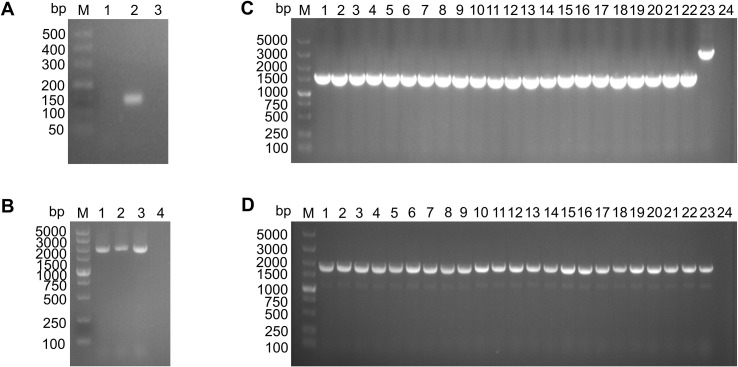
Construction and validation of the △ *iucC* and complementary C-*iucC* strains of *K*. *pneumoniae* KP10. **(A)** PCR detection of △ *iucC*. M: DNA marker (500 bp), Lanes 1: the suspected mutant strain, Lanes 2: the wild-type strain, Lanes 3: negative control. **(B)** PCR verification of the complemented strain C-*iucC.* M: DNA marker (5000 bp), Lanes 1-2: C-*iucC*, Lanes 3: the wild-type strain, Lanes 4: negative control. **(C)** Genetic stability of △ *iucC* (partial). M: DNA marker (5000 bp), Lanes 1–22: △ *iucC*, Lanes 23: the wild-type strain, Lanes 24: negative control. **(D)** Genetic stability of C-*iucC* (partial). M: DNA marker (5000 bp), Lanes 1–22: C-*iucC*, Lanes 23: the wild-type strain, Lanes 24: negative control.

As shown in **Figures 3A, B**, both the △ *iucC* and C-*iucC* strains were successfully constructed. The results of genetic stability determination of the △ *iucC* and C-*iucC* strains indicate that the changes in the genomes of the △ *iucC* and C-*iucC* strains can be stably inherited for more than 50 generations (**Figures 3C, D**). To avoid any polar effects of the *iucC* gene on nearby genes, the relative mRNA expression levels of *iucB* and *iucD* in the wild-type and deletion strains were measured by qRT-PCR. The results showed that the expression of *iucB* and *iucD* was not significantly reduced in the △ *iucC* strain (**Supplementary Figure S2A**, **2B**).

### 3.6 Effects of *iucC* mutation on mucoviscosity

**Figure 4:**
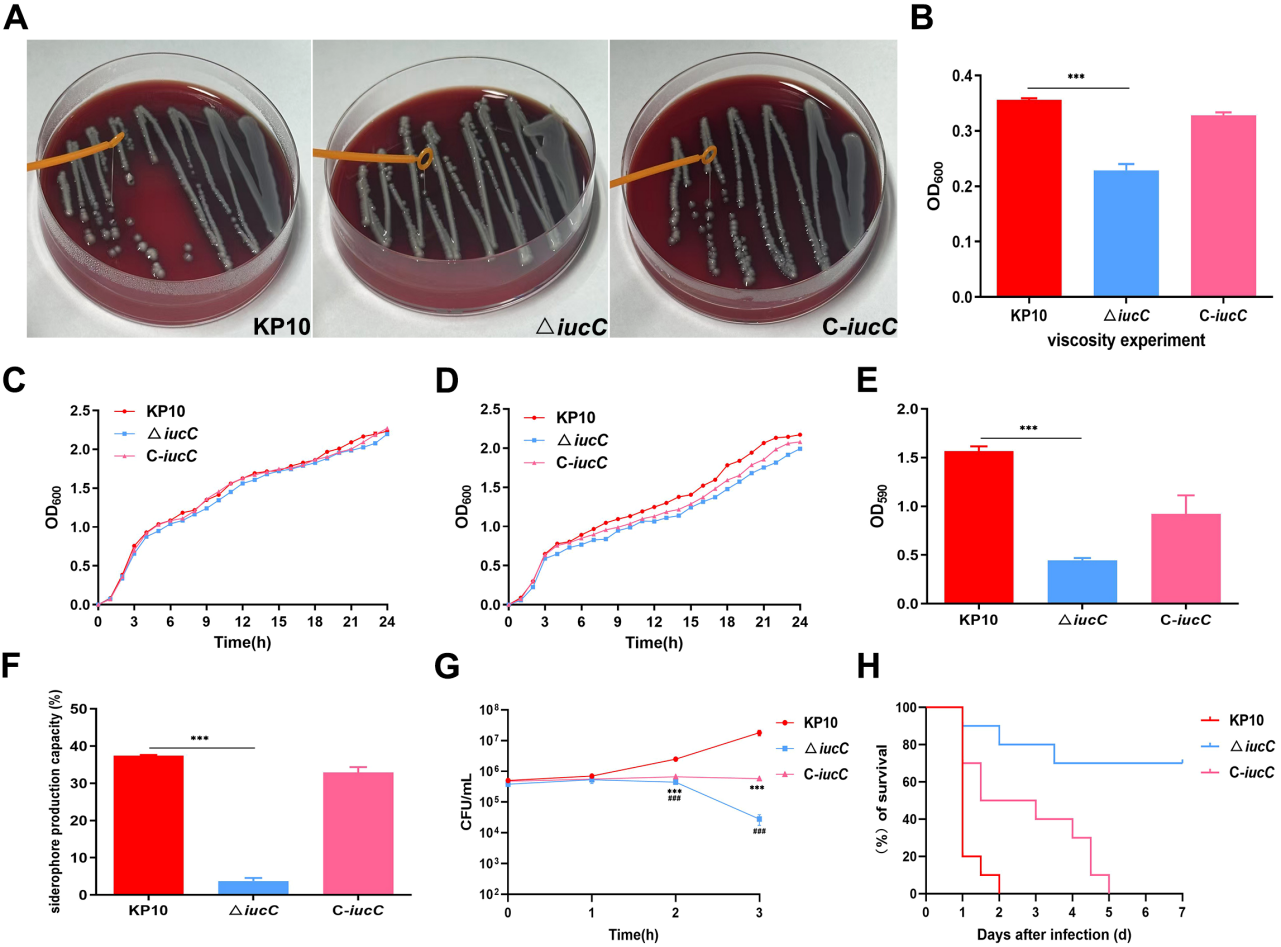
Comparative phenotypic analysis of *K*. *pneumoniae* strains: **(A)** Viscous string of the wild-type KP10, deletion △ *iucC* and complementary C-*iucC* strains. **(B)** Viscosity semiquantitative experiments of the wild-type KP10, deletion △ *iucC* and complementary C-*iucC* strains was determined by the OD_600_ values. ***P <0.001 **(C)** Growth curves of the wild-type KP10, deletion △ *iucC* and complementary C-*iucC* strains under iron-replete conditions was determined by the OD_600_ values. **(D)** Growth curves of the wild-type KP10, deletion △ *iucC* and complementary C-*iucC* strains under iron-limiting conditions. The OD_600_ value was measured by a spectrophotometer at each time point and is displayed in the graph. **(E)** Crystal violet biofilm assays of the wild-type KP10, deletion △ *iucC* and complementary C-*iucC* strains were performed to determine the OD_590_ values. ***P <0.001. **(F)** The ability of the wild-type KP10, deletion △ *iucC* and complementary C-*iucC* strains to produce siderophores was determined by the OD_630_ values. ***P<0.001. **(G)** Differences in serum resistance between the wild-type KP10, deletion △ *iucC* and complementary C-*iucC* strains. ***P<0.001 indicates a comparison between KP10 and C-*iucC*. ###*P*<0.001 indicates a comparison between KP10 and △ *iucC*. **(H)** Survival curves of mice intraperitoneally inoculated with 1 × 10^5^ CFUs of the wild-type KP10, deletion △ *iucC* and complementary C-*iucC* strains. Data are shown as the means ± SDs, n=3.

In the string test, compared with that of *iucC*, the viscosity of the △ *iucC* strain decreased. In contrast, the viscosity of C-*iucC* increased, similar to that of the wild-type strain *iucC*. (**Figure 4A**). Viscosity semiquantitative experiments revealed that the suspension of the KP10 strain was more difficult to settle after low-speed centrifugation, and the viscosity of the KP10 strain was significantly greater than that of the △ *iucC* strain (*P* ≤ 0.001), while the viscosity of the C-*iucC* strain recovered to the level of that of the wild-type strain (**Figure 4B**).

### 3.7 Measurement of bacterial growth assay

The growth curves revealed that the growths of WT, △ *iucC* and C-*iucC* were essentially the same under iron-replete conditions (**Figure 4C**). Under iron-limiting conditions, the growth rate of the wild-type strain was faster than that of the △ *iucC* strain, but the difference was not significant (**Figure 4D**). Compared with that of the △ *iucC* strain, the growth rate of the C-*iucC* strain, recovered but did not reach the level of the wild-type strain.

### 3.8 Roles of *iucC* deletion in biofilm-forming activity

As shown in **Figure 4E**, compared with that of wild-type KP10, the ability of bacteria to form biofilms significantly decreased after the *iucC* gene was deleted (*P* ≤ 0.001). However, the biofilm formation ability of the complementary strain was restored. This indicated that the deletion of the *iucC* gene affected the biofilm formation of the strain.

### 3.9 *IucC* is essential for siderophore production

Compared with that of the WT group and the C-*iucC* group, the siderophore production capacity of the △ *iucC* group significantly decreased (*P* ≤ 0.001), while the siderophore production capacities of the WT and C*-iucC* groups did not change significantly (**Figure 4F**). These results demonstrate that mutation of the *iucC* gene can affect the siderophore production capacity of bacteria.

### 3.10 Influence of the *iucC* mutants on serum resistance

In the serum resistance assay, the survival rate of the △ *iucC* strain was significantly lower than that of the WT strain, and the serum sensitivity of the former was intermediate (**Figure 4G**). The complementation of *iucC* restored the survival rate and resulted in serum resistance. These results confirm the crucial role of *iucC* in resistance to the complement-mediated killing of *K. pneumoniae*.

### 3.11 Toxicity testing *in vivo*

Our results revealed that when the bacteria were injected at doses of 10^5^ CFU/per mouse, all the mice in the wild-type strain treatment group died within 2 days and all the mice in the complementary strain C-*iucC* treatment group died within 5 days (**Figure 4H**). On day 7, the death rate in the △ *iucC* group was 30%. These data indicate that the deletion of *iucC* significantly attenuates the virulence of *K. pneumoniae*.

### 3.12 Bacterial load in blood and tissues

**Figure 5:**
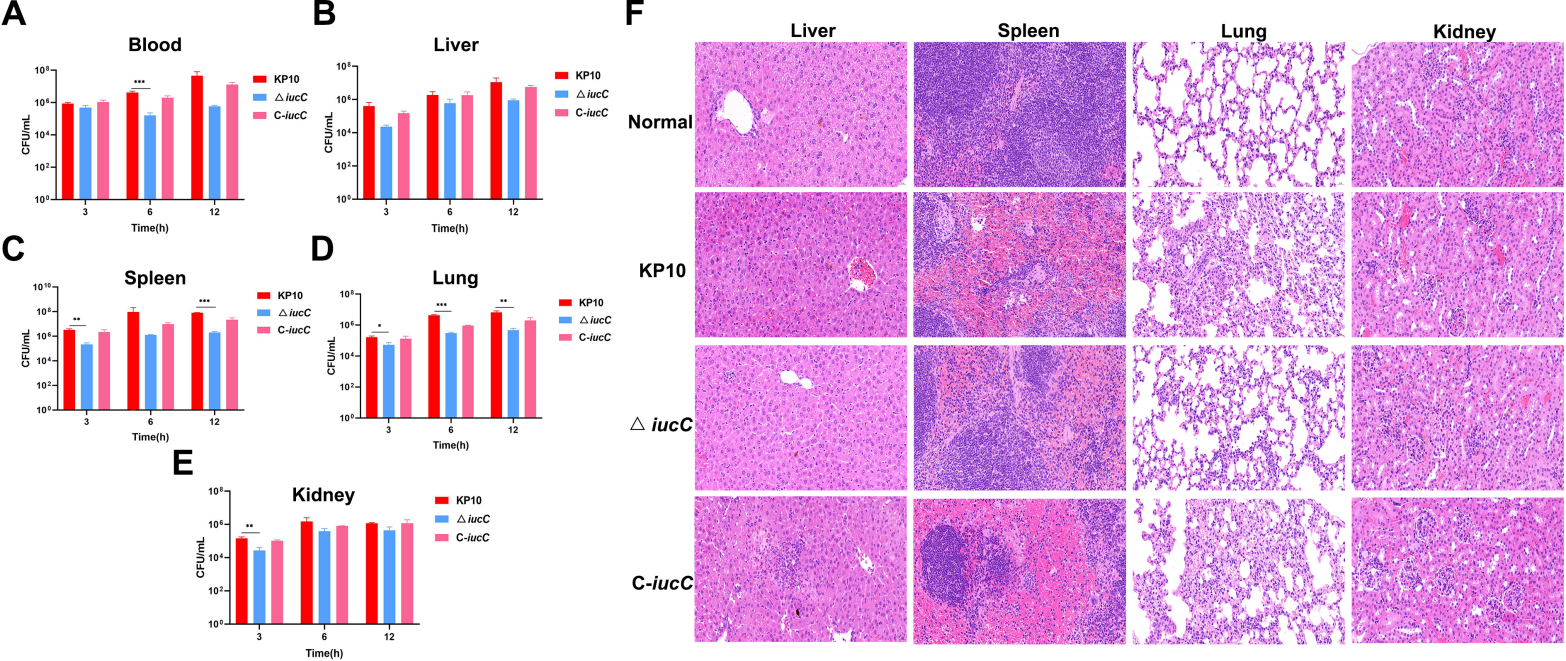
Comparative phenotypic analysis of *K*. *pneumoniae* strains: **(A–E)** Bacterial load in blood and organ samples from mice. Blood and organ samples were collected from each group at 3, 6 and 12 h after the challenge. The bacterial loads of the blood **(A)**, liver **(B)**, spleen **(C)**, lung **(D)** and kidney **(E)**. *P <0.05, **P <0.01, ***P <0.001. The bacterial load was determined on the basis of the colony counts. The values represent the means and SDs (n=3). **(F)** Histopathology of liver, spleen, lung, and kidney in mice were taken and stained with H&E (magnification: ×400) after intraperitoneal infection for 12 h.

After 6 h of infection with the wild-type strains, the bacterial load in the blood of the mice reached approximately 4.2 × 10^6^ CFU/mL. In contrast, the bacterial load in the blood of the mice in the △ *iucC* group significantly decreased (*P* ≤ 0.001), with a bacterial load of approximately 1.6 × 10^5^ CFU/mL. There was no significant difference between the C-*iucC* group mice and the wild-type strain group mice (**Figure 5A**). In addition, after 3 h of infection with wild-type *K. pneumoniae*, the bacterial loads in the liver, spleen, lungs, and kidneys of the mice reached approximately 4 × 10^5^, 3.4 × 10^6^, 1.7 × 10^5^, and 1.5 × 10^5^ CFU/mL, respectively (**Figures 5B-E**). In contrast, the bacterial load in the liver, spleen, lungs, and kidneys of mice infected with △ *iucC* strain decreased significantly, with bacterial loads of approximately 2.3 × 10^4^, 2.2 × 10^5^, 5.3 × 10^4^, and 2.7 × 10^4^ CFU/mL, respectively. There was no significant difference in the bacterial load in various tissues of mice infected with C-*iucC* strain compared with those in the wild-type group.

### 3.13 Histopathological observation

As shown in **Figure 5F**, compared with the healthy group, mice infected with wild-type *K. pneumoniae* or the complementary strain C-*iucC* exhibited varying degrees of pathological changes in the liver, spleen, lungs and kidney, such as hepatic steatosis and hepatocellular necrosis in the liver; atrophy of the germinal center and necrosis of splenocytes in the spleen; inflammatory cellular infiltration in the lungs; and cellular necrosis, inflammatory cellular infiltration and other pathologies in the kidneys. In contrast, although mice infected with the △ *iucC* strain showed different degrees of pathological changes in the liver, spleen, lungs and kidney organs compared with the organs of the healthy group, the changes were not as severe as those in the wild-type and C-*iucC* strain groups.

### 3.14 Effects of the *iucC* gene on the cell adhesion and cytotoxicity of *K. pneumoniae*

**Figure 6:**
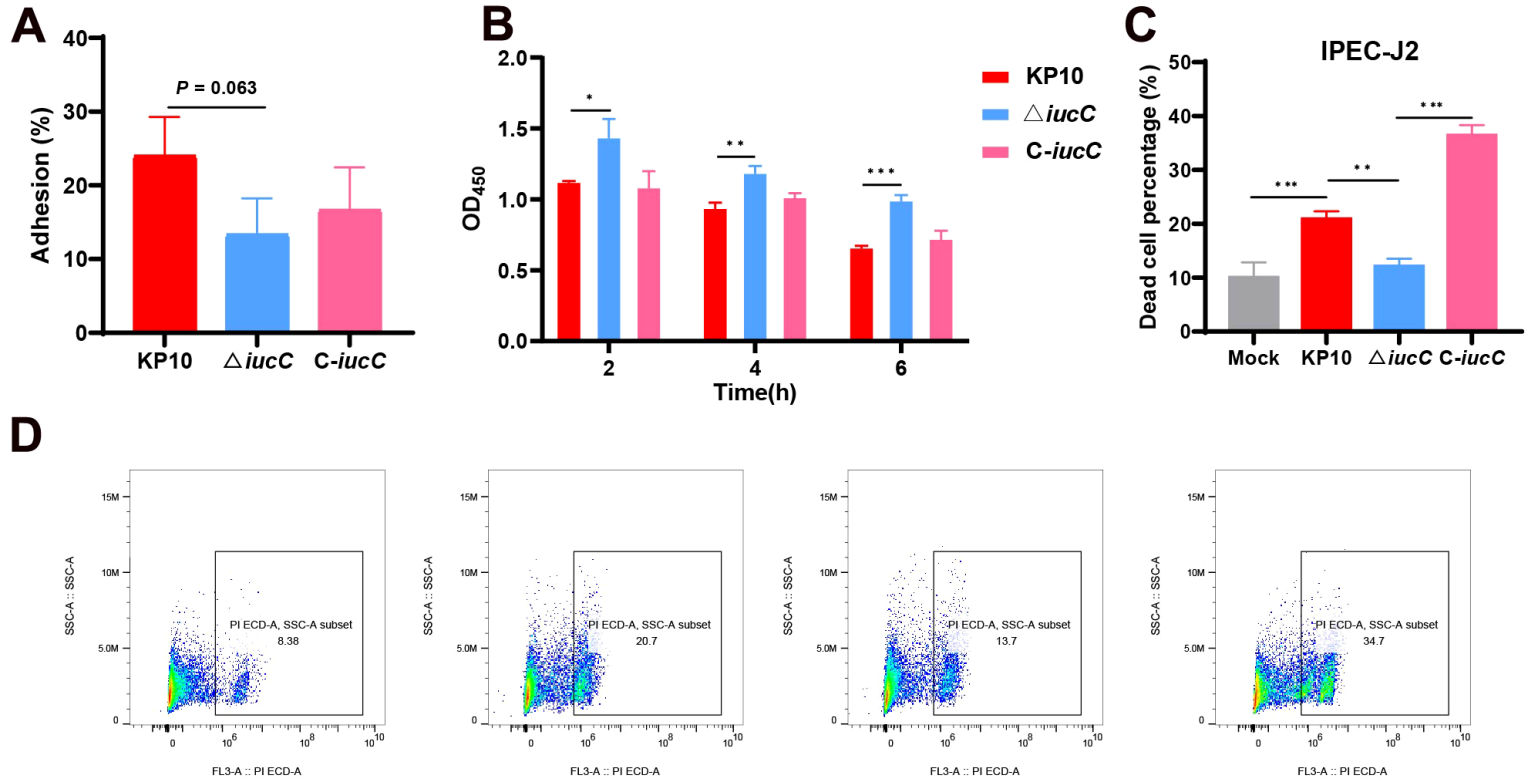
Comparative phenotypic analysis of *K. pneumoniae* strains: **(A)** IPEC-J2 cell adhesion numbers of the wild-type KP10, △ *iucC*, and complementary C-*iucC* strains. **(B)** The cytotoxicity of the wild-type, mutant and complementary strains on IPEC-J2 cells was determined by the OD_450_ values. *P <0.05, **P <0.01, ***P <0.001. **(C)** Survival of the wild-type KP10, △ *iucC*, and complementary C-*iucC* strains. *P <0.05, **P <0.01, ***P <0.001. **(D)** The cell viability rates of the control, wild-type KP10, △ *iucC*, and complementary C-*iucC* strains were tested by flow cytometry. The x-axis represents PIECD-H, and the y-axis represents SSC-H. Data are shown as the means ± SDs, n=3.

The results shown in **Figure 6A** indicate that compared with the wild-type strain, the deletion of the *iucC* gene decreased the total amount of adhesion of *K. pneumoniae* to IPEC cells, whereas the total amount of adhesion to IPEC cells increased in the C-*iucC* strain but did not return to the wild-type level. The *iucC* gene contributes to the adhesion of *K. pneumoniae* to intestinal epithelial cells. In addition, compared with that of the wild-type strain KP10, the toxicity of the deficient strain △ *iucC* to IPEC cells was significantly (*P*<0.05) reduced after 2 h of infection, while the toxicity of the C-*iucC* strain was similar to that of the wild-type strain (**Figure 6B**), indicating that the *iucC* gene is a virulence factor of *K. pneumoniae* KP10. Flow cytometric analysis following PI staining revealed that infection with the △ *iucC* strain induced significantly lower levels of cell death than infection with the wild-type strain did. Notably, this reduced cell death phenotype was reversed in the C*-iucC* strain (**Figures 6C, D**).

### 3.15 Analysis of the evolutionary relationships of the *iucC* gene

**Figure 7:**
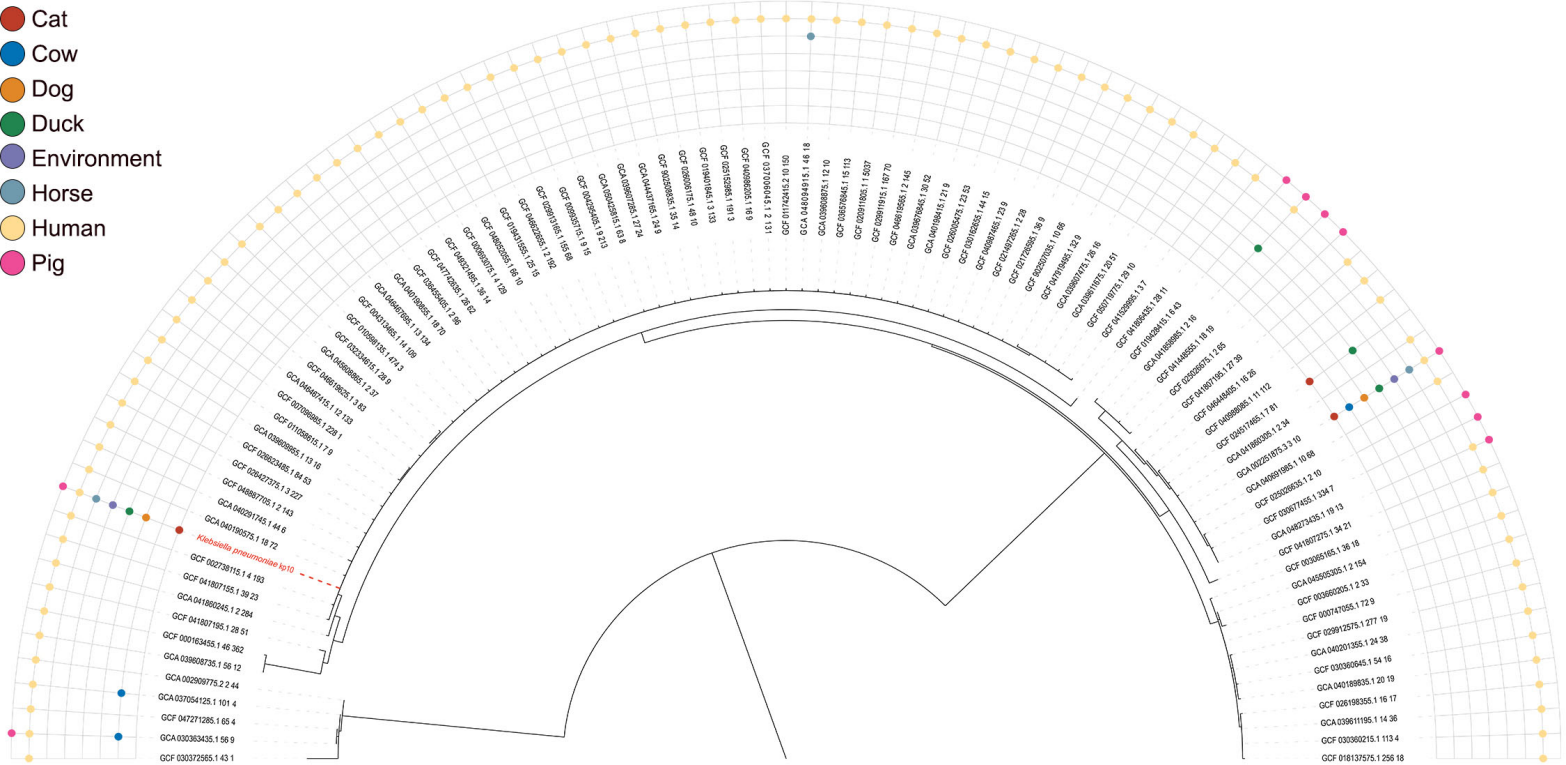
A phylogenetic tree of different genotypes of *iucC* was constructed, where genotypes are defined on the basis of nucleotide sequences differing by at least one base. The outer rings display points in different colours representing isolate sources, including cats, cows, dogs, ducks, the environment, horses, humans, and pigs.

This study investigated the *iucC* gene by analyzing a vast collection of genomes from both laboratory and public sources (30,759 in total). The *iucC* gene was found in 7,732 (25.1%) of the *K. pneumoniae* genomes (100% identity), which originated from humans, 6 different animals (pig, cat, cow, dog, duck and horse) and the environment. The vast majority of gene sequences exhibit significant similarity. Notably, these *K. pneumoniae* strains are primarily derived from humans, followed by pigs. Some *iucC* genes are shared among *K. pneumoniae* from both humans and pigs, while some *iucC* genes are shared among *K. pneumoniae* from humans, six different animals, and environmental sources (**Figure 7**). These findings indicate that *K. pneumoniae* carrying the *iucC* gene may be widely transmitted to humans, animals and the environment.

## 4 Discussion

In recent years, the prevalence of hv*Kp* infection in Asian Pacific Rim has been steadily increasing and hv*Kp* has emerged as a concerning global pathogen (Du et al., 2021). Although the pathogenic mechanism of hv*Kp* has been widely studied, these studies are limited mainly to human derived *K. pneumoniae* (Heng et al., 2024). Considering the zoonotic characteristics of *K. pneumoniae*, the pathogenic mechanism of hv*Kp* from animal sources cannot be ignored. In this study, we investigated the pathogenic factors of hv*Kp* from pigs.

The *khe* gene, which has been used to characterize clinical *K. pneumoniae*, encodes a ‘unique’ haemolysin of *K. pneumoniae* CMC-1 and is considered a potential species-specific gene probe (Yin-Ching et al., 2002). We identified 20 strains of *K. pneumoniae* from pig fecal samples by *khe* gene and 16S rRNA gene identification. Sequencing of the wzi gene is considered a rapid method for capsular genotyping of *K. pneumoniae*, with up to 95% ability to predict the capsular type (K-type) on the basis of the wzi gene sequence (Wu et al., 2022). Our results demonstrated that the wzi gene was sequenced in 20 K*. pneumoniae* isolates, and 11 different serotypes were obtained. Among these, the KP10 and KP6 strains belonged to the K2 serotype. Furthermore, through a string test and toxicity testing in mice, it was determined that KP10 is a hv*Kp*, whereas KP6 has weaker toxicity.

Comparative genomics enables the study of gene selection, mutation, recombination, and genomic function at the genetic level and plays a crucial role in exploring the phylogenetic relationships among bacteria and their genetic evolutionary patterns (Lin et al., 2023). Whole-genome sequencing results revealed that the KP10 genome carries a complete aerobactin operon, which is located on the virulence plasmid. Notably, we specifically detected the virulence related gene *iucC* in KP10, and successfully constructed the △ *iucC* and C-*iucC* strains via homologous recombination. Zi et al. similarly performed the deletion and complementation of genes using this method (Zi et al., 2023), which proved to be more efficient and could be used for the deletion and complementation of *K. pneumoniae*-related genes. And the relative mRNA expression levels of *iucB* and *iucD* were determined by qRT-PCR in the wild-type strain and the deletion mutant. The results showed that the expression of *iucB* and *iucD* was not significantly reduced in the △ *iucC* mutant, suggesting that the deletion of *iucC* did not cause an obvious polar effect on its nearby genes. The results of the growth curves revealed that the growth of the deletion and wild-type strains was essentially the same under iron-complete conditions, whereas the growth of the wild-type strain was faster than that of the △ *iucC* strain under iron-restricted conditions. These findings are similar to those of Karen et al., who reported that the growth of the △ t*onB* and △ *tonB*/△ *fur* mutants was impaired under iron-restricted conditions but not in iron-replete media (Holden et al., 2014). We speculate that deletion of the *iucC* gene results in the inability of the microorganisms to take up iron, leading to a degree of growth inhibition under iron-limiting conditions but not under iron-replete conditions. The string test is used to screen for hv*Kp* phenotypes and has been used as a criterion for determining that *K. pneumoniae* is a high mucus phenotype; however, differences in mucoviscosity, depending on the material stretching the colony, may cause false-negatives in the string test, and therefore viscosity semiquantitative experiments are needed to further determine the hv*Kp* phenotype (Itoga et al., 2024). The mucoviscosity measurements demonstrated that the *iucC* gene affected the mucus phenotype of the KP10 strain. Bacterial biofilms are communities of microorganisms that attach to the surface of the environment and to host surfaces.

The presence of biofilms allows bacteria to resist attack by the host immune system, moreover, *K. pneumoniae* has a strong biofilm-forming capacity, which is one of the reasons for its high virulence. Our data indicated that the *iucC* gene plays an important role in biofilm formation. However, Li et al. reported that aerobactin-mediated iron acquisition contributes to only the biofilm formation of *Y. pseudotuberculosis* under iron-limiting conditions (Li et al., 2021). We speculate that deletion of the *iucC* gene blocks aerobactin synthesis and affects the ability to acquire iron, altering biofilm formation. CAS is a universally applicable method for detecting the production of siderophores. Our results show that deletion of the *iucC* gene leads to reduced siderophore production. This may be because the lack of *iucC* affects the function of siderophores, leading to an insufficient supply of iron and a decrease in the amount of siderophores secreted by the bacteria, which affects the amount of siderophores secreted in the CAS assay. Our results are strikingly similar to the findings of Han et al., who reported that △ *entB* and △ *ybtS* lead to decreased secretion of siderophores (Han et al., 2022). Serum contains a variety of antibodies and complement components that recognize and bind to the bacterial surface, thus activating the complement system and triggering a number of immune responses that ultimately destroy the bacteria. Therefore, serum resistance tests can be used to determine the anti-killing ability of bacteria. Our work revealed that the deletion strains became serum sensitive. Federica et al. reported that *tonB3* deletion increased *A. baumannii* susceptibility to normal human serum (Runci et al., 2019).

In this study, using a bacteraemia model, we revealed that the ability of the △ *iucC* strain to cause mouse death and maintain bacterial loads in the blood, liver, spleen, kidneys, and lungs were impaired. Further histopathological examination demonstrated that △ *iucC* attenuated pathological damage to the liver, spleen, kidney and lung. The diminished bacterial loads, pathological changes and pathogenicity explained the attenuation of the virulence of △ *iucC* in mice. These results collectively revealed that *iucC* is a major contributor to virulence in the KP10 hv*Kp* strain. Ling et al. reported that *iucC* plays important roles in the pathogenicity of APEC E058, as shown in our results (Ling et al., 2013). The initial step of pathogen infection involves adhering to the surface of host cells, with epithelial cells being a primary target. This adhesion is a vital first step in the infection process and is essential for the pathogen to successfully invade host cells (Hsieh et al., 2019). We demonstrate here that compared with the wild-type strain, the deletion of *iucC* results in reduced cell adhesion, cytotoxicity, and survival. This may be because deletion of the *iucC* gene leads to impaired iron uptake, which indirectly affects bacterial metabolism and growth, thus diminishing its ability to adhere to host cells and its cytotoxicity. According to existing literature, *iroB*, *iucA*, *peg-344*, *rmpA*, and *rmpA2* are the five key virulence marker factors that define hvKp, and all of these markers have been shown to exist on virulence plasmids (Russo and Marr, 2019). In this study, the *iucC* gene was also present on the virulence plasmid. After the *iucC* gene was knocked out, the virulence of Klebsiella pneumoniae was significantly reduced, indicating that the *iucC* gene is also a major contributor to virulence of hv*Kp*, which may assist in enhancing the virulence of the strain.

Lam et al. demonstrated that plasmids carrying the iuc locus (which includes *iucC*) could be horizontally transferred between different clones, including the hv*Kp* clonal group CG23 (Lam et al., 2018). To investigate the distribution and evolution of the *iucC* gene, we analyzed 30,758 public genomes from NCBI alongside 20 newly sequenced porcine *K. pneumoniae* strains in this study. Notably, the *iucC* gene was found in genomes from humans and six diverse animal and environmental sources, suggesting potential horizontal transmission between hosts. This finding indicates that *K. pneumoniae* encoding *iucC* is widely present, plays an important role in the pathogenicity of the strain, and may undergo extensive horizontal transfer. These findings suggest that we need to increase our attention to the virulence factor *iucC* and deepen our research on its pathogenic mechanism. This gene may be used as a target to develop vaccines against hv*Kp*, which is the goal of our future work.

## 5 Conclusion

In this study, we successfully obtained K2 serotype *K. pneumoniae* strains of varying virulence from porcine hosts. Through comparative genomic analysis, the virulence-associated gene *iucC* was identified in the high-virulence strain KP10, and functional analysis using an *iucC* deletion mutant (△ *iucC*) and complemented strains (C-*iucC*) revealed the critical role of *iucC* in multiple virulence-associated mechanisms, including biofilm formation, siderophore production, serum resistance, adhesion, cytotoxicity, and ultimately virulence in murine models. Genetic evolution analysis revealed that the *iucC* gene is widely present in animals and humans, indicating that *K. pneumoniae* carrying the *iucC* gene may be widely transmitted in humans, animals and the environment. These findings provide a crucial theoretical foundation for elucidating *iucC* function and the pathogenesis of *K. pneumoniae.*

## Data Availability

The datasets presented in this study can be found in online repositories. The names of the repository/repositories and accession number(s) can be found below: https://www.ncbi.nlm.nih.gov/, JBRBRF000000000 https://www.ncbi.nlm.nih.gov/, JBRBRB000000000 https://www.ncbi.nlm.nih.gov/, JBRBQW000000000 https://www.ncbi.nlm.nih.gov/, JBRBQX000000000 https://www.ncbi.nlm.nih.gov/, JBRBQY000000000 https://www.ncbi.nlm.nih.gov/, JBRBQZ000000000 https://www.ncbi.nlm.nih.gov/, JBRBRA000000000 https://www.ncbi.nlm.nih.gov/, JBRBRC000000000 https://www.ncbi.nlm.nih.gov/, JBRBRD000000000 https://www.ncbi.nlm.nih.gov/, JBRBRE000000000 https://www.ncbi.nlm.nih.gov/, JBRBRG000000000 https://www.ncbi.nlm.nih.gov/, JBRBRH000000000 https://www.ncbi.nlm.nih.gov/, JBRBRI000000000 https://www.ncbi.nlm.nih.gov/, JBRBRJ000000000 https://www.ncbi.nlm.nih.gov/, JBRBRK000000000 https://www.ncbi.nlm.nih.gov/, JBRBRL000000000 https://www.ncbi.nlm.nih.gov/, JBRBRM000000000 https://www.ncbi.nlm.nih.gov/, JBRBRN000000000 https://www.ncbi.nlm.nih.gov/, JBRBRO000000000 https://www.ncbi.nlm.nih.gov/, JBRBRP000000000.

## References

[B1] Aboulela A. El-Sherbini E. Abu-Sheasha G. El-Raouf H. A. El-Ghazzawi E. Gaballah A. (2020). Molecular strain typing of multidrug-resistant *Klebsiella pneumoniae*: capsular *wzi* gene sequencing versus multiple locus variable number tandem repeat analysis. Diagn. Microbiol. Infect. Dis. 98, 115139. doi: 10.1016/j.diagmicrobio.2020.115139, PMID: 32861156

[B2] Al-Busaidi B. Al-Muzahmi M. Al-Shabibi Z. Rizvi M. Al-Rashdi A. Al-Jardani A. . (2024). Hypervirulent Capsular Serotypes K1 and K2 *Klebsiella pneumoniae* Strains Demonstrate Resistance to Serum Bactericidal Activity and Galleria mellonella Lethality. Int. J. Mol. Sci. 25, 1944. doi: 10.3390/ijms25031944, PMID: 38339222 PMC10855873

[B3] Arato V. Raso M. M. Gasperini G. Berlanda Scorza F. Micoli F. (2021). Prophylaxis and Treatment against *Klebsiella pneumoniae*: Current Insights on This Emerging Anti-Microbial Resistant Global Threat. Int. J. Mol. Sci. 22, 4042. doi: 10.3390/ijms22084042, PMID: 33919847 PMC8070759

[B4] Bailey D. C. Alexander E. Rice M. R. Drake E. J. Mydy L. S. Aldrich C. C. . (2018). Structural and functional delineation of aerobactin biosynthesis in hypervirulent *Klebsiella pneumoniae*. J. Biol. Chem. 293, 7841–7852. doi: 10.1074/jbc.RA118.002798, PMID: 29618511 PMC5961048

[B5] Bidewell C. A. Williamson S. M. Rogers J. Tang Y. Ellis R. J. Petrovska L. . (2018). Emergence of *Klebsiella pneumoniae* subspecies pneumoniae as a cause of septicaemia in pigs in England. PloS One 13, e0191958. doi: 10.1371/journal.pone.0191958, PMID: 29470491 PMC5823397

[B6] Choby J. E. Howard-Anderson J. Weiss D. S. (2020). Hypervirulent *Klebsiella pneumoniae* - clinical and molecular perspectives. J. Intern. Med. 287, 283–300. doi: 10.1111/joim.13007, PMID: 31677303 PMC7057273

[B7] Du L. Zhang J. Liu P. Li X. Su K. Yuan L. . (2021). Genome sequencing and comparative genome analysis of 6 hypervirulent *Klebsiella pneumoniae* strains isolated in China. Arch. Microbiol. 203, 3125–3133. doi: 10.1007/s00203-021-02263-0, PMID: 33811489 PMC8019302

[B8] Edwards B. S. Ivnitski-Steele I. Young S. M. Salas V. M. Sklar L. A. (2007). High-throughput cytotoxicity screening by propidium iodide staining. Curr. Protoc. Cytom. Chapter 9, Unit9, 24. doi: 10.1002/0471142956.cy0924s41, PMID: 18770858

[B9] Feldman M. F. Mayer Bridwell A. E. Scott N. E. Vinogradov E. McKee S. R. Chavez S. M. . (2019). A promising bioconjugate vaccine against hypervirulent *Klebsiella pneumoniae*. Proc. Natl. Acad. Sci. U S A. 116, 18655–18663. doi: 10.1073/pnas.1907833116, PMID: 31455739 PMC6744904

[B10] Fu Y. Ni P. Zhang Y. Liang F. Stover N. A. Li L. (2024). The genome and comparative transcriptome of the euryhaline model ciliate *Paramecium duboscqui* reveal adaptations to environmental salinity. BMC Biol. 22, 237. doi: 10.1186/s12915-024-02026-5, PMID: 39407207 PMC11476214

[B11] Gu D. Dong N. Zheng Z. Lin D. Huang M. Wang L. . (2018). A fatal outbreak of ST11 carbapenem-resistant hypervirulent *Klebsiella pneumoniae* in a Chinese hospital: a molecular epidemiological study. Lancet Infect. Dis. 18, 37–46. doi: 10.1016/S1473-3099(17)30489-9, PMID: 28864030

[B12] Han R. Niu M. Liu S. Mao J. Yu Y. Du Y. (2022). The effect of siderophore virulence genes *entB* and *ybtS* on the virulence of Carbapenem-resistant *Klebsiella pneumoniae*. Microb. Pathog. 171, 105746. doi: 10.1016/j.micpath.2022.105746, PMID: 36064103

[B13] He Y. Guo X. Xiang S. Li J. Li X. Xiang H. . (2016). Comparative analyses of phenotypic methods and 16S rRNA, *khe*, *rpoB* genes sequencing for identification of clinical isolates of *Klebsiella pneumoniae*. Antonie Van Leeuwenhoek 109, 1029–1040. doi: 10.1007/s10482-016-0702-9, PMID: 27147066

[B14] Heng H. Yang X. Ye L. Tang Y. Guo Z. Li J. . (2024). Global genomic profiling of *Klebsiella pneumoniae*: A spatio-temporal population structure analysis. Int. J. Antimicrob. Agents 63, 107055. doi: 10.1016/j.ijantimicag.2023.107055, PMID: 38081547

[B15] Hetland M. A. K. Winkler M. A. Kaspersen H. P. Håkonsholm F. Bakksjø R. J. Bernhoff E. . (2025). A genome-wide One Health study of *Klebsiella pneumoniae* in Norway reveals overlapping populations but few recent transmission events across reservoirs. Genome Med. 17, 42. doi: 10.1186/s13073-025-01466-0, PMID: 40296028 PMC12039103

[B16] Holden K. M. Browning G. F. Noormohammadi A. H. Markham P. Marenda M. S. (2014). Avian pathogenic *Escherichia coli* Δ*tonB* mutants are safe and protective live-attenuated vaccine candidates. Vet. Microbiol. 173, 289–298. doi: 10.1016/j.vetmic.2014.07.028, PMID: 25205199

[B17] Holden V. I. Breen P. Houle S. Dozois C. M. Bachman M. A. (2016). *Klebsiella pneumoniae* Siderophores Induce Inflammation, Bacterial Dissemination, and HIF-1α Stabilization during Pneumonia. mBio 7, e01397–e01316. doi: 10.1128/mBio.01397-16, PMID: 27624128 PMC5021805

[B18] Holden V. I. Wright M. S. Houle S. Collingwood A. Dozois C. M. Adams M. D. . (2018). Iron acquisition and siderophore release by carbapenem-resistant sequence type 258 *klebsiella pneumoniae*. mSphere 3, e00125–e00118. doi: 10.1128/mSphere.00125-18, PMID: 29669884 PMC5907654

[B19] Hou G. Ahmad S. Li Y. Yan D. Yang S. Chen S. . (2024). Epidemiological, virulence, and antibiotic resistance analysis of *klebsiella pneumoniae*, a major source of threat to livestock and poultry in some regions of Xinjiang, China. Anim. (Basel) 14, 1433. doi: 10.3390/ani14101433, PMID: 38791650 PMC11117231

[B20] Hsieh P. F. Hsu C. R. Chen C. T. Lin T. L. Wang J. T. (2016). The *Klebsiella pneumoniae YfgL* (*BamB*) lipoprotein contributes to outer membrane protein biogenesis, type-1 fimbriae expression, anti-phagocytosis, and *in vivo* virulence. Virulence 7, 587–601. doi: 10.1080/21505594.2016.1171435, PMID: 27029012 PMC5038167

[B21] Hsieh P. F. Lu Y. R. Lin T. L. Lai L. Y. Wang J. T. (2019). *Klebsiella pneumoniae* type VI secretion system contributes to bacterial competition, cell invasion, type-1 fimbriae expression, and *in vivo* colonization. J. Infect. Dis. 219, 637–647. doi: 10.1093/infdis/jiy534, PMID: 30202982 PMC6350951

[B22] Hu D. Chen W. Wang W. Tian D. Fu P. Ren P. . (2023). Hypercapsule is the cornerstone of *Klebsiella pneumoniae* in inducing pyogenic liver abscess. Front. Cell Infect. Microbiol. 13. doi: 10.3389/fcimb.2023.1147855, PMID: 37065211 PMC10102340

[B23] Hu D. Chen W. Zhang Q. Li M. Yang Z. Wang Y. . (2022). Prevalence of Carbapenem-Resistant Hypervirulent *Klebsiella pneumoniae* and Hypervirulent Carbapenem-Resistant *Klebsiella pneumoniae* in China Determined via Mouse Lethality Tests. Front. Cell Infect. Microbiol. 12. doi: 10.3389/fcimb.2022.882210, PMID: 35719357 PMC9199425

[B24] Ibrahim G. A. Salah-Eldein A. M. Al-Zaban M. I. El-Oksh A. S. A. Ahmed E. M. Farid D. S. . (2023). Monitoring the genetic variation of some *Escherichia coli* strains in wild birds and cattle. J. Vet. Res. 90, e1–e10. doi: 10.4102/ojvr.v90i1.2085, PMID: 37526530 PMC10483432

[B25] Itoga M. Hayashi W. Kayama S. Yu L. Sugawara Y. Kimura M. . (2024). Severe co-infection caused by difficult-to-diagnose hypermucoviscous *Klebsiella pneumoniae* K1-ST82 in a patient with COVID-19: a case report. BMC Infect. Dis. 24, 1215. doi: 10.1186/s12879-024-10092-x, PMID: 39468457 PMC11520518

[B26] Lam M. M. C. Wyres K. L. Duchêne S. Wick R. R. Judd L. M. Gan Y. H. . (2018). Population genomics of hypervirulent *Klebsiella pneumoniae* clonal-group 23 reveals early emergence and rapid global dissemination. Nat. Commun. 9, 2703. doi: 10.1038/s41467-018-05114-7, PMID: 30006589 PMC6045662

[B27] Lee C. R. Lee J. H. Park K. S. Jeon J. H. Kim Y. B. Cha C. J. . (2017). Antimicrobial resistance of hypervirulent *klebsiella pneumoniae*: epidemiology, hypervirulence-associated determinants, and resistance mechanisms. Front. Cell Infect. Microbio. 7. doi: 10.3389/fcimb.2017.00483, PMID: 29209595 PMC5702448

[B28] Lei T. Y. Liao B. B. Yang L. R. Wang Y. Chen X. B. (2024). Hypervirulent and carbapenem-resistant *Klebsiella pneumoniae*: A global public health threat. Microbiol. Res. 288, 127839. doi: 10.1016/j.micres.2024.127839, PMID: 39141971

[B29] Li L. Li F. Hu X. Wu Z. Ren W. Wang T. . (2022). LAP3 contributes to IFN-γ-induced arginine depletion and Malignant transformation of bovine mammary epithelial cells. BMC Cancer 22, 864. doi: 10.1186/s12885-022-09963-w, PMID: 35941558 PMC9358085

[B30] Li C. Pan D. Li M. Wang Y. Song L. Yu D. . (2021). Aerobactin-mediated iron acquisition enhances biofilm formation, oxidative stress resistance, and virulence of *yersinia pseudotuberculosis*. Front. Microbiol. 12. doi: 10.3389/fmicb.2021.699913, PMID: 34335534 PMC8319957

[B31] Li X. S. Qi Y. Xue J. Z. Xu G. Y. Xu Y. X. Li X. Y. . (2023). Transcriptomic Changes and *satP* Gene Function Analysis in *Pasteurella multocida* with Different Levels of Resistance to Enrofloxacin. Vet. Sci. 10, 257. doi: 10.3390/vetsci10040257, PMID: 37104412 PMC10143902

[B32] Lin Y. Lu J. Yang Z. Wang T. Li H. Sha S. . (2023). Comparative genomics reveals key molecular targets for mutant *Pediococcus pentosaceus* C23221 producing pediocin. Int. J. Biol. Macromol. 242, 125006. doi: 10.1016/j.ijbiomac.2023.125006, PMID: 37224904

[B33] Ling J. Pan H. Gao Q. Xiong L. Zhou Y. Zhang D. . (2013). Aerobactin synthesis genes *iucA* and *iucC* contribute to the pathogenicity of avian pathogenic *Escherichia coli* O2 strain E058. PloS One 8, e57794. doi: 10.1371/journal.pone.0057794, PMID: 23460907 PMC3584046

[B34] Mendes G. Santos M. L. Ramalho J. F. Duarte A. Caneiras C. (2023). Virulence factors in carbapenem-resistant hypervirulent *Klebsiella pneumoniae*. Front. Microbiol. 14. doi: 10.3389/fmicb.2023.1325077, PMID: 38098668 PMC10720631

[B35] Muner J. J. de Oliveira P. A. A. Baboghlian J. Moura S. C. de Andrade A. G. de Oliveira M. M. . (2024). The transcriptional regulator *Fur* modulates the expression of *uge*, a gene essential for the core lipopolysaccharide biosynthesis in *Klebsiella pneumoniae*. BMC Microbiol. 24, 279. doi: 10.1186/s12866-024-03418-x, PMID: 39061004 PMC11282780

[B36] Pu D. Zhao J. Chang K. Zhuo X. Cao B. (2023). Superbugs” with hypervirulence and carbapenem resistance in *Klebsiella pneumoniae*: the rise of such emerging nosocomial pathogens in China. Sci. Bull. (Beijing) 68, 2658–2670. doi: 10.1016/j.scib.2023.09.040, PMID: 37821268

[B37] Runci F. Gentile V. Frangipani E. Rampioni G. Leoni L. Lucidi M. . (2019). Contribution of active iron uptake to *acinetobacter baumannii* pathogenicity. Infect. Immun. 87, e00755–e00718. doi: 10.1128/IAI.00755-18, PMID: 30718286 PMC6434119

[B38] Russo T. A. Gulick A. M. (2019). Aerobactin synthesis proteins as antivirulence targets in hypervirulent *klebsiella pneumoniae*. ACS Infect. Dis. 5, 1052–1054. doi: 10.1021/acsinfecdis.9b00117, PMID: 31032610 PMC6625901

[B39] Russo T. A. Marr C. M. (2019). Hypervirulent *klebsiella pneumoniae*. Clin. Microbiol. Rev. 32, e00001–19. doi: 10.1128/CMR.00001-19, PMID: 31092506 PMC6589860

[B40] Russo T. A. Olson R. Fang C. T. Stoesser N. Miller M. MacDonald U. . (2018). Identification of Biomarkers for Differentiation of Hypervirulent *Klebsiella pneumoniae* from Classical *K. pneumoniae*. J. Clin. Microbiol. 56, e00776–e00718. doi: 10.1128/JCM.00776-18, PMID: 29925642 PMC6113484

[B41] Shao L. Yao B. Yang J. Li X. Ye K. Zhang Y. . (2020). Characterization of a multidrug-resistant *Klebsiella pneumoniae* ST3330 clone responsible for a nosocomial outbreak in a neonatal intensive care unit. Ann. Palliat Med. 9, 1092–1102. doi: 10.21037/apm-20-958, PMID: 32434364

[B42] Walker K. A. Miner T. A. Palacios M. Trzilova D. Frederick D. R. Broberg C. A. . (2019). A *Klebsiella pneumoniae* Regulatory Mutant Has Reduced Capsule Expression but Retains Hypermucoviscosity. mBio 10, e00089–e00019. doi: 10.1128/mBio.00089-19, PMID: 30914502 PMC6437046

[B43] Wang Y. D. Gong J. S. Guan Y. C. Zhao Z. L. Cai Y. N. Shan X. F. (2023). *OmpR* (TCS response regulator) of *Aeromonas veronii* plays a major role in drug resistance, stress resistance and virulence by regulating biofilm formation. Microb. Pathog. 181, 106176. doi: 10.1016/j.micpath.2023.106176, PMID: 37244492

[B44] Wang G. Zhao G. Chao X. Xie L. Wang H. (2020). The characteristic of virulence, biofilm and antibiotic resistance of *klebsiella pneumoniae*. Int. J. Environ. Res. Public Health 17, 6278. doi: 10.3390/ijerph17176278, PMID: 32872324 PMC7503635

[B45] Wu Z. Li N. Li Z. Wang J. Liu M. Qi M. . (2023). Development and application of an indirect ELISA and nested PCR for the epidemiological analysis of *Klebsiella pneumoniae* among pigs in China. Front. Microbiol. 14. doi: 10.3389/fmicb.2023.1329609, PMID: 38260894 PMC10803024

[B46] Wu X. Liu J. Feng J. Shabbir M. A. B. Feng Y. Guo R. . (2022). Epidemiology, environmental risks, virulence, and resistance determinants of *klebsiella pneumoniae* from dairy cows in Hubei, China. Front. Microbiol. 13. doi: 10.3389/fmicb.2022.858799, PMID: 35602033 PMC9117759

[B47] Wu Y. Yang Y. Dang H. Xiao H. Huang W. Jia Z. . (2021). Molecular identification of *Klebsiella pneumoniae* and expression of immune genes in infected spotted gar Lepisosteus oculatus. Fish Shellfish Immunol. 119, 220–230. doi: 10.1016/j.fsi.2021.10.002, PMID: 34626790

[B48] Yang Y. Higgins C. H. Rehman I. Galvao K. N. Brito I. L. Bicalho M. L. . (2019b). Genomic diversity, virulence, and antimicrobial resistance of *klebsiella pneumoniae* strains from cows and humans. Appl. Environ. Microbiol. 85, e02654–e02618. doi: 10.1128/AEM.02654-18, PMID: 30610074 PMC6414388

[B49] Yang B. Song H. An D. Zhang D. Raza S. H. A. Wang G. . (2019a). Functional Analysis of *preA* in *Aeromonas veronii* TH0426 Reveals a Key Role in the Regulation of Virulence and Resistance to Oxidative Stress. Int. J. Mol. Sci. 21, 98. doi: 10.3390/ijms21010098, PMID: 31877791 PMC6981600

[B50] Yang X. Sun Q. Li J. Jiang Y. Li Y. Lin J. . (2022). Molecular epidemiology of carbapenem-resistant hypervirulent *Klebsiella pneumoniae* in China. Emerg. Microbes Infect. 11, 841–849. doi: 10.1080/22221751.2022.2049458, PMID: 35236251 PMC8942559

[B51] Yin-Ching C. Jer-Horng S. Ching-Nan L. Ming-Chung C. (2002). Cloning of a gene encoding a unique haemolysin from *Klebsiella pneumoniae* and its potential use as a species-specific gene probe. Microb. Pathog. 33, 1–6. doi: 10.1006/mpat.2002.0499, PMID: 12127794

[B52] Zhu L. Li P. Zhang G. He Z. Tao X. Ji Y. . (2023). Role of the ISKpn element in mediating *mgrB* gene mutations in ST11 hypervirulent colistin-resistant *Klebsiella pneumoniae*. Front. Microbiol. 14. doi: 10.3389/fmicb.2023.1277320, PMID: 37840706 PMC10569121

[B53] Zi C. Yang S. Fu X. Wang W. Luo Y. Zhang J. . (2023). An efficient method for knocking out genes on the virulence plasmid of hypervirulent *Klebsiella pneumoniae*. New Microbiol. 46, 186–195., PMID: 37247239

