# Supplementary material for: The iucC gene is a major contributor to the virulence of hypervirulent Klebsiella pneumoniae
Source: Front Cell Infect Microbiol. 2026 Jan 12;15:1742219. doi: 10.3389/fcimb.2025.1742219 (PMC12832671; doi:10.3389/fcimb.2025.1742219)
Supplement: Supplementary file 1 [file DataSheet1.docx]

# Supplementary Figures and Tables


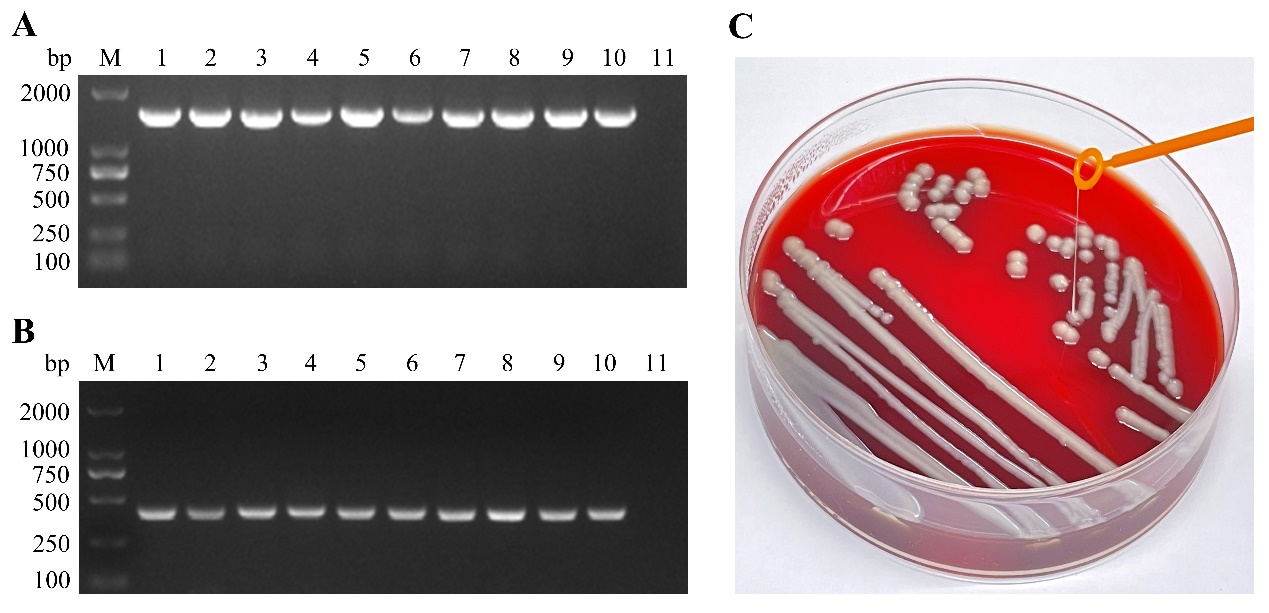


Figure S1. Isolation and identification of *K. pneumoniae*. (A) PCR amplification of 16S rDNA (partial). M: DNA marker (2000 bp), Lanes 1–10: PCR product of the isolated strains KP1-KP10, Lanes 11: negative control. Specific bands of approximately 1500 bp, corresponding to the expected size of the 16S rDNA fragment were successfully amplified from all isolates, while no band was observed in the negative control, confirming the absence of contamination. (B) PCR amplification of *khe* gene (partial). M: DNA Marker (2000 bp), Lanes 1–10: PCR product of the isolated strains KP1-KP10, Lanes 11: negative control. Specific bands of approximately 428 bp, corresponding to the expected size of the *khe* gene fragment, were successfully amplified from all isolates. No band was observed in the negative control, confirming the absence of contamination. (C) The results of the string test. The viscous strings of KP10 and KP11 were positive, measuring >5 mm; the viscous strings of the other strains were negative, measuring <5 mm.


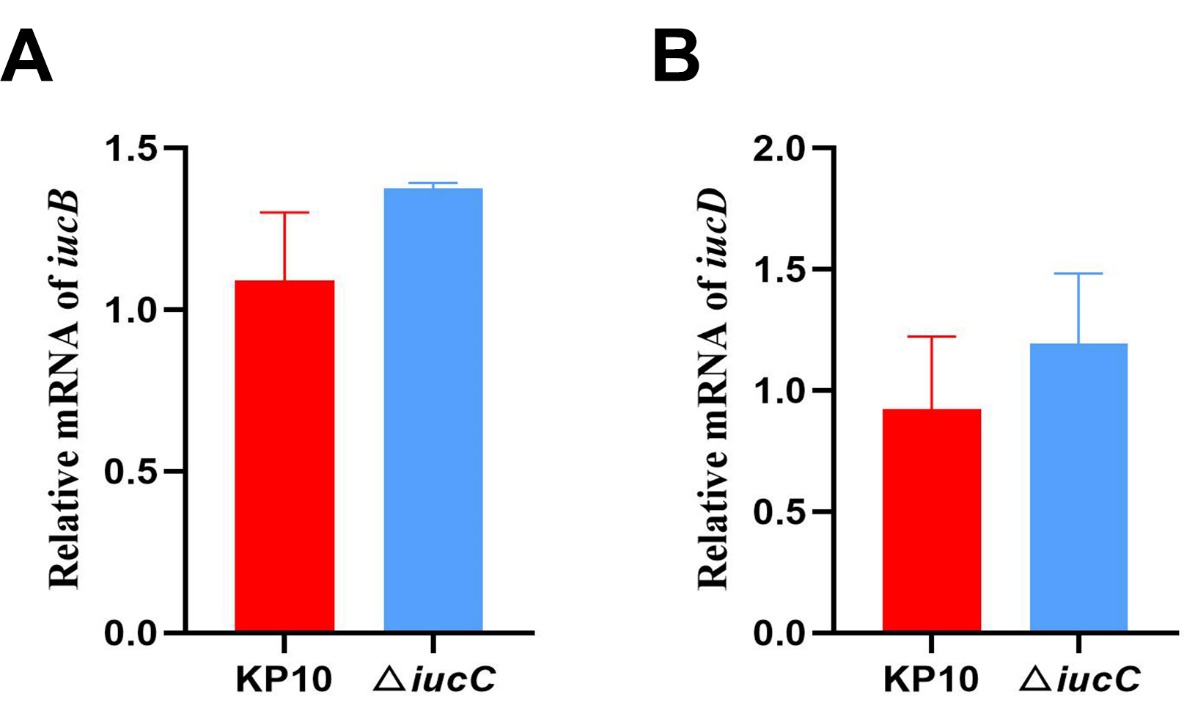


Figure S2. Relative expression levels of mRNA for *iucB* and *iucD* genes using qRT-PCR. (A) The mRNA expression levels of *iucB* in the KP10, and △ *iucC* strains. (B) The mRNA expression levels of *iucD* in the KP10, and △ *iucC* strains. Data are shown as the means ± SDs, n = 3.

Table S1. Primers used for PCR amplifications.

| Primer name | Primer sequences (5′-3′) | Product size (bp) | Annealing temperature (℃) |
| --- | --- | --- | --- |
| 27 F | AGAGTTTGATCTGGCTCAG | 1500 | 55 |
| 1492 R | GGTTACCTTGTTACGACTT |  |  |
| *khe*-F | TGATTGCATTCGCCACTGG | 428 | 55 |
| *khe*-R | GGTCAACCCAACGATCCTG |  |  |
| *Wzi*-F | GTGCCGCGAGCGCTTTCTATCTTGGTATTCC | 600 | 61 |
| *Wzi*-R | GAGAGCCACTGGTTCCAGAACTTCACCGC |  |  |
| *iucC*-up-F (*Xba*I) | GCTCTAGATGCCGTCGGAGGTCATTTTCTG | 817 | 63 |
| *iucC*-up-R | ATGAATCACAAGGCAACCAGGGACTGAATC |  |  |
| *iucC*-down-F | TCAGTCCCTGGTCCAATCCTTGTGATTCAT | 820 | 63 |
| *iucC*-down-R (*Sac*I) | ATGAGCTCTATTTATCGCCGCACCGCAACTT |  |  |
| pRE112-F | GCGATGAGTGGCAGGGC |  | 61 |
| pRE112-R | TTACCGACTGCGGCCTGAGT |  |  |
| *iucC*-I-F | TCCATCAGTGTTGCCATCAGAATCG | 143 | 59 |
| *iucC*-I-R | GGCTATGTGTCCCATGAAGGCTATG |  |  |
| *iucC*-up-F | GC TCTAGA TGCCGTCGGAGGTCATTTTCTG | 1637 | 63 |
| *iucC*-down-R | ATGAGCTCTATTTATCGCCGCACCGCAACTT |  |  |
| *iucC* -P-F (*Xba*I) | GCTCTAGAGCAGATTGTTCATCCCTTC | 2093 | 58 |
| *iucC* -P-R (*Sac*I) | ATGAGCTCATGAATCACAAGGATTGGG |  |  |
| pBBR-F | TAAGTTGGGTAACGCCAGG |  | 54 |
| pBBR-R | GAGTTAGCTCACTCATTAGGC |  |  |
| *iucC*-C-F | TCAGTCCCTGGTTGCCAG | 1734 | 51 |
| *iucC*-C-R | ATGAATCACAAGGATTGGG |  |  |
| *iucB*-F | CCGCTTTGCTCCCAGAAATAC | 105 | 62 |
| *iucB*-R | TCGCAAGACGCTGAGTTTCC |  |  |
| *iucD*-F | ACTCAGGCGTGAAGTATTCGTTG | 124 | 62 |
| *iucD*-R | CTGTTTCTCAATGCGCTACGC |  |  |
| 16S rRNA-F | ACTCCTACGGGAGGCAGCAG | 197 | 55 |
| 16S rRNA-R | ATTACCGCGGCTGCTGG |  |  |
